# An exploratory study to assess the activity of the acarine growth inhibitor, fluazuron, against *Sarcoptes scabei *infestation in pigs

**DOI:** 10.1186/1756-3305-5-40

**Published:** 2012-02-16

**Authors:** Cielo Pasay, Jim Rothwell, Kate Mounsey, Andrew Kelly, Beverly Hutchinson, Alon Miezler, James McCarthy

**Affiliations:** 1 Clinical Tropical Medicine Laboratory, Queensland Institute of Medical Research (QIMR), 300 Herston Road, Herston, QLD 4006; 2 School of Veterinary Science, University of Queensland, Gatton Campus, Gatton, QLD 4343; 3 Currently: Meat and Livestock Australia, 165 Walker St, North Sydney, NSW, 2060; 4 Centre for Advanced Animal Science (CAAS) DEEDI: Department of Employment, Economic Development and Innovation located at University of Queensland, Gatton Campus, Gatton, Qld; 5 School of Medicine, University of Queensland, Herston, QLD 4006; 6 School of Health and Sports Sciences, University of Sunshine Coast, QLD 4556

**Keywords:** scabies alternative treatment, acarine growth inhibitor, fluazuron

## Abstract

**Background:**

The most common treatments for scabies in human and veterinary settings are topical 5% permethrin or systemic treatment with ivermectin. However, these treatments have very little activity against arthropod eggs, and therefore repeated treatment is frequently required. *In-vitro*, biochemical and molecular studies have demonstrated that human mites are becoming increasingly resistant to both acaricides. To identify alternate acaricides, we undertook a pilot study of the *in vivo *activity of the benzoylphenyl urea inhibitor of chitin synthesis, fluazuron, in pigs with sarcoptic mange.

**Findings:**

Pigs (n = 5) were infested with *S. scabei *var *suis*, and randomised to treatment at the start of peak infestation with fluazuron at a dose of 10 mg/kg/day *per os *for 7 days (n = 3) or no treatment (n = 2). Clinical scores, skin scrapings for mite counts and blood sampling for pharmacokinetic analysis were undertaken. Fluazuron was well absorbed in treated pigs with measureable blood levels up to 4 weeks post treatment. No adverse effects were observed. Modest acaricidal activity of the compound was observed, with a reduction in severity of skin lesions in treated pigs, as well as a reduction in number of scabies mite's early life stages.

**Conclusions:**

The moderate efficacy of fluazuron against scabies mites indicates a lead to the development of alternate treatments for scabies, such as combination therapies that maybe applicable for human use in the future.

## Findings

Scabies is an infectious skin disease caused by a microscopic mite, *Sarcoptes scabiei*. The number of treatments employed for this parasitic disease is very limited. The most common treatments in human and veterinary settings are topical 5% permethrin and/or systemic treatment with a macrocyclic lactone, such as ivermectin [1]. These acaricides have relatively little activity against arthropod eggs, hence multiple treatments are generally required to achieve cure. Using *in-vitro*, biochemical and molecular approaches, we have previously demonstrated that mites are becoming increasingly resistant to both acaricides [2-4]. The emergence of acaricide resistance in mites has highlighted the need to identify new acaricidal agents. We have previously investigated the acaricidal activity of natural product extracts of tea-tree oil and clove oil and their active components using *in vitro *studies of mites collected from pigs with experimental scabies infestation [5]. These studies have shown potential useful acaricidal activity of essential oils- tea-tree (*Melaleuca alternifola*) and clove (*Eugenia caryophyllata*), and their active components [6,7].

Another approach to the development of acaricides suitable for human use is to investigate the utility of existing veterinary products with likely acaricidal activity. Commercially available veterinary acaricides include Fipronil and Amitraz. These are effective treatments for Sarcoptic mange in dogs and cats. Both disrupt function of the nervous system of arthropods and cause a knock down effect similar to that of pyrethroids and organochloride insecticides. Benzoylphenyl urea (BPU) compounds act as acaricides in a different way, by blocking the synthesis and deposition of chitin, thereby preventing molting and sterilising eggs [8,9]. Earlier studies have shown that BPU compounds are active at low concentrations against arthropods and have the potentially useful property of a long-lasting, residual effect [10-12]. Importantly they have high specificity, with low mammalian toxicity [13].

Fluazuron is a BPU acarine growth inhibitor that is marketed for the control of cattle tick, *Rhipicephalus microplus*. As this compound inhibits specific enzymes involved in moulting, drug-exposed immature ticks are unable to moult to the next stage, and treated females lay sterile eggs [14]. Flurazuron has also been used to control fleas on wild rodents, woodrats and mice [15], and as an oral/systemic acaricide to control ticks and fleas of woodrats [16]. We conducted an exploratory trial to assess the activity of orally administered fluazuron against *Sarcoptes scabei *infestation in pigs.

## Methods

The study was approved by the Animal Ethics Committee (Approval number: SA 2010/11/335). Three-week female, weaner pigs (*Sus scrofa*) were infected with *S. scabiei var suis *maintained on a daily 0.2 mg/kg dexamethasone as previously described [5] to enhance infestation intensity. Prior to treatment, fortnightly skin scrapings were performed to monitor mite number; the relative numbers of different life stages were assessed during the course of infestation. Skin crusting and lesions associated with mite infestation were also monitored on a weekly basis using a scoring system that we had developed in a previously published study [5]: Scores of 1-8, where 1-3 indicates acute, papular mange; 4 and above indicates crusts of increasing severity [5]. As no relevant data were available to inform a power calculation for assigning study size, for welfare and logistical reasons a study size of six was set, with three pigs assigned to the treatment and three to the control (untreated) group. One piglet died unexpectedly of an unrelated cause before the trial commenced. Hence, the remaining five pigs were randomly allocated to treatment (n = 3) and untreated control (n = 2) groups. The treatment and control pigs were housed in separate rooms (in individual pens) of the experimental animal holding facility of the Centre for Advanced Animal Studies (CAAS) at UQ, Gatton to prevent cross contamination. Fluazuron was administered as an oral drench using a syringe simultaneously to the treatment group beginning 8 weeks following mite infestation. At this point, skin lesions were clinically evident in all trial pigs (Clinical scores of 1-4). Baseline mite counts prior to treatment were also obtained for each trial pig. An equal amount of skin crusts (~ 0.15 grams), from skin lesions on the back and ears of the trial pigs were collected, and the number and life stages of mites were recorded. Commercially formulated fluazuron (Acatak^® ^Novartis Animal Health, Macquarie Park, NSW) containing 25 mg/ml fluazuron was given daily by mouth for 7 days at 0.4 ml/kg to give 10 mg/kg/day to the treatment group. A 7-day regimen was selected for this first study as the investigators were seeking to identify an effect at a high dose regimen that could subsequently be optimised. Blood samples (10 ml) were collected for pharmacokinetic analysis 24 hours before treatment (Day 0) and on Days 1, 2, 5, 7, 21 and 28 post treatment. Serum levels of fluazuron were determined by HPLC-UV (Waters Instrumentation, Ireland) using an in-house fluazuron standard calibration curve. Skin scrapings were collected from trial pigs, 2 and 7 weeks post treatment to monitor mite status, numbers and life stages. Skin crusting and lesions in all trial pigs were also scored at the same time intervals. Pigs were euthanased at the end of the study. No statistical inferences were made because of limitations in sampling from a small number of trial pigs.

## Results

**Figure 1:**
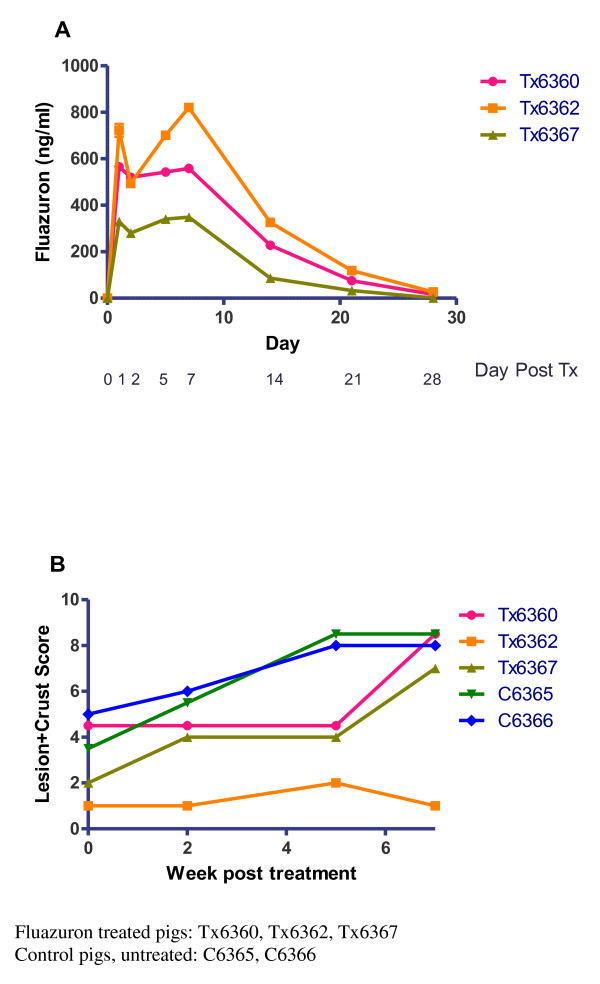
**(A) Fluazuron blood levels in trial pigs and (B) Clinical presentation of trial pigs**.

Fluazuron was quickly absorbed in three treated pigs, with a rapid rise in blood levels 24 hours after oral administration. Blood levels of fluazuron remained detectable for 28 days after initiation of treatment (Figure 1A).

Clinical progression of the disease (in terms of lesion + crust scores) continued to rise from baseline (week 0) in the control, untreated pigs (C6365 and C6366) reaching a maximum at week 5 onwards, continuing to week 8 when the study was concluded. In contrast, progression of clinical score in the treated pigs was arrested up to week 5 post treatment. However, after week 5, relapse of the clinical symptoms of scabies in pigs Tx6360 and Tx6367 was observed. Disease progression was totally abrogated in Tx6362 pig. Of note, this pig had the highest blood level of fluazuron (Figure 1B).

**Figure 2:**
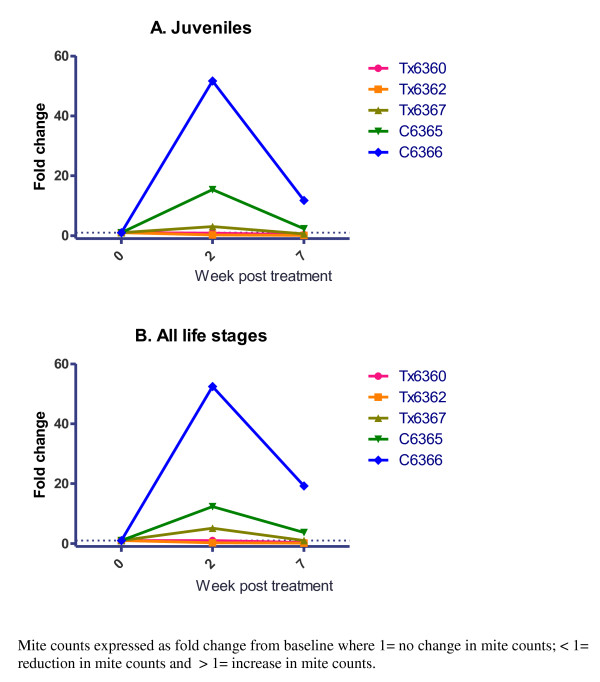
**Fold-change in mite counts in trial pigs in (A) Juveniles and (B) All life stages**.

From 2 weeks post treatment, a decline in overall mite counts especially in early life stages was observed in two fluazuron treated pigs (Tx6360 and Tx6362). This reduction is expressed as a fold change of < 1 compared to the baseline mite counts (set at 1) (Figure 2A and 2B). Tx6367 pig which had the lowest level of blood fluazuron showed the slowest reduction in juvenile stage mite counts with changes observed only on the 7th week post treatment. Of note, the baseline mite burden of this pig was heavy and the oral dosage (10 mg/kg) used may not have been sufficient to kill all mites present in the crusted skin. In comparison, the untreated pigs had an increase (> 1 fold change) in mite counts compared to baseline from 2 weeks onwards.

Trial pigs were euthanased at the conclusion of the study and no adverse effects of oral administration of fluazuron was observed post mortem.

## Discussion

Ivermectin is the only oral drug available to treat human scabies. As it does not have activity against eggs, repeated treatment is generally required, with the second dose generally administered after 7-10 days [1]. A single dose regimen that would target all life stages to achieve complete cure would represent a major advantage. Fluazuron represents a potential partner to ivermectin; it works well with cattle ticks and targets the parasite's early life stages. In a similar study, a mixture of a juvenile hormone regulator (pyriproxyfen) and a bioinsecticide (spinosad), has demonstrated effective control of insecticide resistant *Aedes aegypti *[17].

As it is not possible to test the effect of Fluazuron against the mite eggs *in vitro*, we conducted this *in-vivo *trial in the mange pig model. We observed an effect on the population structure of mites, with a decrease in numbers of juvenile stages evident from 2 weeks post treatment of trial pigs. Overall moderate efficacy was observed, with arrest of clinical progression of scabies in treated pigs until week 5. Although complete clearance of lesions was not observed, a notable decrease in mite counts was achieved (post treatment compared to baseline) especially in juvenile stages in treated pigs. No adverse effects of fluazuron on the pigs were observed at necropsy examination.

The oral dose rate of 10 mg/kg/day resulted in peak blood levels of 300 to 800 ng/mL (300 - 800 ug/L) fluazuron. This blood level in pigs is significantly higher than those achieved in cattle following either pour-on treatment (35 - 41 ug/L) or following subcutaneous administration (100 ug/L) at a dose of 1.5 mg/kg [18]. However, in this study, the higher plasma level of fluazuron resulted in modest acaricidal activity, without total clearance of the skin lesions. Unlike ticks, mites do not ingest blood, and thus may have been exposed to suboptimal fluazuron levels. This modest activity may also be related to the *in vivo *pig model used (maintained on dexamethasone), which results in higher mite numbers and more severe lesions than would be observed in a natural infestation of scabies.

This exploratory study is the first *in-vivo *study to be conducted to test acaricidal activity of an acarine growth inhibitor (fluazuron) in scabies. This investigation paves the way for studies aimed at exploring alternate treatments for scabies, such as combination therapies, for example, combination studies with drugs acting at different lifecycle stages. Such combinations may form the basis of a treatment strategy applicable for human use in the future.

## Competing interests

The authors declare that they have no competing interests.

## Authors' contributions

CP conceived and designed the study, collected samples, performed mite counts, analysed data, drafted the manuscript. JR participated in study conception and design, treatment of trial pigs, collected blood samples, facilitated pharmacokinetic studies, performed post mortem examination of trial pigs, edited manuscript. KM participated in study design, infestation of trial pigs, collection of samples, data analysis, edited manuscript. AK and BH participated in study design, facilitated treatment of trial pigs and collection of samples, performed clinical scorings of trial pigs, performed animal facility duties related to trial. AM performed the pharmacokinetic analysis of blood samples from trial pigs. JM participated in design and conception of study, analysis and interpretation of data. All authors reviewed and approved the manuscript.
